# Back to Actual Behavior – A Modest Proposal on the Example of Exploratory Behavior in Children on the Autism Spectrum

**DOI:** 10.1007/s12124-023-09763-2

**Published:** 2023-07-18

**Authors:** Ewa Pisula, Wojciech Pisula

**Affiliations:** 1 Faculty of Psychology, University of Warsaw, Stawki 5/7, 00-183 Warsaw, Poland; 2 Institute of Psychology, PAS, Jaracza 1, 00-378 Warsaw, Poland

**Keywords:** Exploratory Behavior, Ethology, Theory of Integrative Levels, Autism Spectrum, Children

## Abstract

Autism spectrum is characterized by difficulties in social communication and interactions and limited, restricted patterns of behavior, interests, and activity. It is diagnosed and described on the basis of behavioral characteristics. Therefore, behavior research plays a critical role in better understanding the functioning of this group of children. Studies on their interests and curiosity take mainly the form of experiments on visual and object exploration. We argue that important aspects of behavior might be neglected within these studies and propose to refine the approach by incorporating the achievements of classic ethology, contemporary measurement and analytical tools, and the theory of integrative levels. We conclude with an outline of the proposed approach in a short list of major methodological postulates.

## Introduction

Autism is defined as a spectrum of neurodevelopmental disorders (autism spectrum disorders, ASD), characterized by deficits in social communication and social interactions, and limited, restricted patterns of behavior, interests and activity (American Psychiatric Association, 2013; World Health Organization, 2022). Individuals on the autism spectrum experience difficulties in social-emotional reciprocity, adapting behavior to social context, and non-verbal communication (e.g. maintaining eye contact, communicating with facial expressions, gestures or body posture). Diagnostic criteria also include reduced play, in particular its pretend, imitation and symbolic forms. The core aspects of the autism spectrum include repetitive movement patterns, stereotyped use of objects and/or speech, as well as excessive attachment to specific routines or rituals. Interests also tend to be restricted and untypical in terms of subject or intensity. In addition, the diagnostic criteria take into account the specifics of sensory processing.

Despite nearly eight decades of research and progress in the field, we still do not fully understand the etiology of autism. Attempts to identify reliable biomarkers of the autism spectrum have so far not been entirely successful (Jensen et al., 2022) and diagnosis is still based on the analysis of behavior in terms of its atypicality. This fact, coupled with social communication problems and limited insight of individuals on the spectrum into their own states and experiences (Huang et al., 2017), means that behavior-focused research plays a particularly important role in better understanding the functioning of this population.

One of the areas of this research focuses on exploratory behavior, considered a prerequisite for cognitive development (DeLouize et al., 2017), as well as an important element in the process of adaptation to environment (Pisula, 2003). In this present paper we propose an approach inspired by the theoretical framework developed by Berlyne (1962), which provides a comprehensive and context-based analysis of behavior by concentrating on its various manifestations, rather than narrowly defined processes. We draw on classical ethology, as well as the latest advances in behavior measurement and analysis. In order to avoid excessive reductionism in behavior regulation, we propose using Feibleman’s theory of integrative levels (1954). Our conclusions present key notions of a research program that would allow for a more comprehensive, non-reductive, and at the same time objective description of behavior. This approach aligns with the postulates explicitly made by El-Hani and Pereira (2000).

## Berlyne’s Concept as Theoretical Groundwork for the Study of Exploratory Behavior

In the mid-twentieth century there has been a growing interest in cognitive aspects of behavior (Mandler, 2002), which saw the study of exploration as one of the new areas of research. A breakthrough in the development of this new area of knowledge came with the publication of Berlyne’s two works (Berlyne 1962, 1966) where he points out the fact that the organism’s activity takes on different forms, including ones that are spontaneous and uncontrolled by the configuration of stimuli and reinforcements designed by the investigator, as was the case in the paradigm of behavioral psychology dominant at that time. Berlyne (1962, p. 287) offered the best known and widely accepted definition of exploratory behavior which describes it in terms of its function: “Exploratory responses have the function of altering the stimulus field. They modify stimulation from sources that are already represented in the stimulus field, and they introduce stimulation from sources that were not hitherto represented.”

In hindsight, Berlyne emerges as the pioneer of cognitive psychology, since the key element of his analyses was the aspect of behavior regulation by information incoming to the subject. He was the first to distinguish between diversive and specific exploration. Specific exploration concept stands for the “… exploration that is aimed at stimuli coming from one particular source, providing information about one particular object or event…” (Berlyne, 1960, p. 80). Diversive exploration, on the other hand, refers to a situation in which “A person who seeks entertainment, relief from boredom, or new experiences will be satisfied with stimuli from any of a wide range of sources, provided only that their collative properties are just right.” (Berlyne, 1960, p. 80). This laid the foundation for thinking about exploration as a phenomenon caused either by external stimuli (specific exploration) or motivated endogenously (diversive exploration).

The interest in exploratory behavior of animals kindled by the works of Berlyne, as well as Fowler (1963) and Dember and Fowler (1958) contributed to the new wave of research in this field on humans, mostly children (e.g.Henderson & Moore, 1979, 1980; Minuchin, 1971; Ruff et al., 1984). Some of it involved children with developmental problems, including those with autism, whose untypical behavior towards people and objects prompted questions about the characteristics of these adaptively important forms of behavior.

## Research on Exploratory Behavior in Autistic Children then and Now

One of the first researchers to study exploration in children with autism was Corinne Hutt (Hutt, 1967, 1968, 1969, 1970; Hutt & Hutt, 1965). In her work she used the theoretical framework developed by Berlyne (1962, 1966), paying particular attention to specific exploration (Hutt, 1967, 1968, 1969). Hutt investigated this form of exploration by using a purpose-built object. It was a box with a lever which, when manipulated, set off a buzzer or chime. The method was characterized by high ecological validity with respect to this particular form of exploration. According to the terminology used in ecological psychology, the object in her research invited the child to manipulate with it (Withagen et al., 2012). The object’s properties determined the scope of the child’s behavior taken into account and shaped the measurement of behavior, as well as limited the freedom to explore the environment. Researchers were focused on behavior that was object-related mainly, and the wide range of information about the child’s other activities in the experimental setting was likely lost.

The other type of exploratory activity, diverse exploration, was investigated by Hutt and Hutt (1965) in a study whose main objective was to analyze stereotyped behavior. The study group consisted of 6 children presenting with high rates and intensity of such behavior. The children were observed through a one-way screen in four situations: variant A was a waiting-room all the children had been previously — on this occasion it was empty except for fixtures like the sink and light-switches; in situation B a box of colored wooden blocks was placed in the room; in C the blocks were also present and a female adult sat passively in one corner of the room; in D the adult attempted to get the child to build a standard structure with the blocks. According to the researchers premise, situations A, B, C and D were arranged in the order of increasing complexity. The goal was to find out if conditions differing in complexity would be associated with the intensity of stereotyped behavior and to measure exploratory activity in each condition. It was found that children’s stereotyped behaviors increased as a function of environmental complexity, except where there was intervention from the adult. Stereotypy and block-manipulation increased at the expense of body-manipulation and fixture-manipulation. The authors analyzed stereotyped behavior as manifestation of stimulation regulation, which back then was also the commonly approved explanation of stereotypy in animals.

In a publication that summed up her research on exploration in children with autism, Hutt (1970) listed differences between specific vs. diversive exploration (Table 1). The comparison proposed by Hutt built on Berlyne (1962) by indicating the inherent differences between the two types of exploration. While specific exploration was considered to be a process induced by external environmental factors, diversive exploration was mainly associated with internal motivational processes. This distinction was reflected in further research, in which specific exploration was investigated mainly in studies focusing on environmental effects, while diversive exploration became part of research on individual differences.

**Table 1:** Principal characteristics of specific vs. diversive exploration (after Hutt, 1970)

1. Concerns those inspective, investigative responses directed to a particular source of stimulation, i.e. stimulus-oriented	1. Concerns those activities which seem to increase stimulation irrespective of source, i.e., response-oriented
2. Occurs in presence of a highly stimulating (by virtue of novelty, complexity etc.) set of environmental factors	2. Occurs in absence of specific environmental stimulation
3. Consists of consummatory response to stimulus—change	3. Consists of instrumental response for stimulus change
4. Extrinsically motivated	4. Intrinsically motivated
5. Characterized by response stereotypy	5. Characterized by response variability or entropy
6. Occupies superordinate position in motivational hierarchy in that it can inhibit most tissue-preserving activities	6. Low in motivational hierarchy and can be inhibited by almost any other drive state

Hutt’s work on specific exploration contributed to the emergence of research on “object exploration.” For several decades this area of research has been present in work on exploratory behavior both in typically developing children and children on the autism spectrum (Jarvis et al., 2020; McDuffie et al., 2015; Solis et al., 2017; Vig, 2007) or other developmental problems, e.g. Down syndrome (O’Neil and Happe´, F. G. E. 2000). The studies are conducted on children of different age groups, typically infants or toddlers, using a variety of objects and procedures. Their findings paint a complex picture of differences between children diagnosed with ASD or at-risk for ASD and their peers developing typically or diagnosed with other developmental disorders. The results revealed between-group differences in repetitive use of objects, e.g. in terms of behaviors such as spinning or rotating (Jacques et al., 2018; Ozonoff et al., 2008), as well as activities such as grasping, dropping, mouthing and looking (Kaur et al., 2015). The differences identified in that research are linked with the characteristic feature of the autism spectrum, namely restricted and repetitive behaviors. On the other hand, in studies which took into account behavior measures such as frequency and duration of overall object explorations, number of different objects explored, or exploration of specific objects, differences between children on the autism spectrum and age-matched typically developing children did not reach the level of statistical significance (Jacques et al., 2018).

In studies on object exploration, the freedom of children’s exploration is often significantly restricted, e.g. by placing them in a chair with table top upon which the experimental objects were placed (e.g. Kaur et al., 2015). On the one hand, this helps focus the child’s activity on object-based exploration, but on the other it creates artificial conditions preventing her from demonstrating the whole range of behaviors that could be displayed when the child comes into contact with similar, unfamiliar objects.

A slightly different approach to the study of exploration in children with developmental disorders was first adopted by Switzky et al. (1974), who used shapes designed by Munsinger and Kessen (1964). In this method, the stimulus material consisted of three-dimensional plastic polygons differing in complexity as determined by the number of sides (from 3 in the case of a triangle up to 40 sides). Children were able to take objects in hand, mouth them, smell them or rub them with their hands etc., however, possibilities of more complex manipulation were limited. The method used by Switzky et al. (1974) was similar to what is currently referred to as perceptual exploration and used to be called perceptual curiosity (Berlyne, 1950). Recent work in this area has focused mainly on studying visual exploration (e.g. Franchak, 2020; Gustafsson et al., 2022). It features sophisticated technology and accurate measurements, as well as the ability to control for factors that could affect those measurements. However, the main problem is ecological validity. As noted by Apicella and Barrett (2016, p. 95) “Ecological validity refers to the fit between the task and the ecological (natural or ancestral) problem it is supposed to be mimicking, and external validity refers to the task’s ability to yield results that can be generalized beyond the task itself. For abstract and decontextualized tasks, it’s important to consider these factors when contemplating how the findings bear on the underlying hypotheses.” Despite technological advances allowing for, among other things, greater freedom of movement of children during a study, we can hardly conclude that – with respect to highly active children – techniques that use wires or equipment attached to the participant, and often still radically limiting the ability to move about, meet the conditions discussed previously. Focusing on the measurement of only one aspect of exploratory behavior i.e. visual exploration of presented (usually displayed) objects provides information on a small fraction of exploratory activity. If, for example, a cuddly toy is displayed on the screen, the child “explores” it visually, but is unable to investigate the object in any other way, which she would probably do if she had the opportunity. In the end, we learn very little about the child’s exploration aimed at finding out the object’s characteristics and understanding what it is. For instance, we do not know whether the child would use other methods of exploration (e.g. touching, licking, smelling), which could tell us more about her way of dealing with a partly or completely unfamiliar object or environment and the information collected about that environment (Cf. de Campos et al., 2013; Libertus et al., 2014). Granted, these studies provide important information about selective data processing (see Franchak, 2020). However, by only using visual stimuli and concerning visual aspects of response to stimulation, they clearly do not fully measure behavioral responses to affordances that invite behavior (Withagen et al., 2012).

In conclusion, both the research on object exploration and studies conducted within the visual exploration paradigm are fraught with significant restrictions in the scope of observed and measured behavior that are built in by the researchers. Consequently, they fail to capture a more comprehensive picture of exploration as a way of investigating environment, which may put significant limitations on the ability to accurately interpret the child’s behavior. In natural conditions exploration engages the whole organism/subject, rather than individual analyzers or Motor Effectors, i.e. Individual Parts of the Organism.

These briefly discussed lines of inquiry into object and visual exploration provide a lot of valuable information, but most of it is limited to selective processes (see Franchak, 2020). There is no doubt that a more comprehensive approach to behavioral analysis of children on the autism spectrum in diverse situations and environments is lacking. Data from such studies would be particularly useful in the context of designing strategies to support autistic children, such as creating environmental settings that would facilitate development while taking into account the specifics of their functioning. A concept worth considering in this respect would be the ethological approach.

### Ethological Approach in Research on Exploration

In the first half of the twentieth century, a new paradigm based on ethology emerged in the study of animal and human behavior, which Tinbergen (1963) defined as the biological study of behavior. Typically, ethology is understood as the scientific study of animal and human behavior, usually with a focus on behavior under natural conditions, and viewing behavior as an evolutionarily adaptive phenomenon. This approach treats behavior as a whole, which means that all of its components are viewed as parts of a larger whole, e.g. entire chains of behavior.

A holistic analysis of behavior is possible, for example, by using a classical research method developed in animal ethology, namely the ethogram. In its simplest form, an ethogram is an exhaustive list of all behaviors exhibited by individuals. Each behavior is operationally defined, meaning that it is defined by the criteria that must be satisfied before that behavior can be said to have occurred (Renner, 2018). The use of ethological methods to study the behavior of humans (including children) has a long and tumultuous history (Odenwald, 2022). In the 1960s and 1970s there were many fascinating attempts to study human behavior using methods developed in research on animals. These studies lead to the founding of a new sub-discipline called human ethology. The date of its establishment is widely considered to be the year of publication of the seminal work “Hate and Love” (Eibl-Eibesfeldt, 1971). Unfortunately, it coincided with the wave of fierce disputes associated with social changes, leading to the criticism of the use of terms such as “natural behavior” or “instinct” (Cf. Moltz, 1965; Ridley, 1995, p. 107). As a consequence, many scientists working in the human ethology paradigm at the time abandoned these terms. At the same time, rapid advances in laboratory techniques in the following decades allowed for stricter controls of some factors affecting behavior and more precise measurements of behavior in well-controlled conditions. Focusing on narrower aspects of a phenomenon as complex as behavior in specific environmental conditions made research easier to conduct and delivered more publishable data in shorter amount of time. This fact may also have contributed to significant decline in popularity of the ethological approach to the study of behavior.

There are likely multiple reasons for the abandonment of ethological methods of analyzing human behavior. However, the resulting gap in research protocols may significantly reduce the validity of human behavior analyses compared with a holistic and context-based approach. Table 2 presents a comparison of some features of the experimental-laboratory and ethological methods.

**Table 2:** Characteristics of experimental vs. ethological methods

Characteristics	Experimental/Lab methods	Ethological methods
Variables measured	Precisely defined and selected; limited number, limited domain	Long list of behavioral activities measured; not limited in number and domain
How behavior is analyzed	Behavior is perceived through the lens of particular methodology; limited to the narrow domain, specific to the measures adopted in the study; data about low/fundamental mechanisms of behavior are collected	Behavior is perceived in the ecological context in a holistic way; behavior patterns are extracted, and adaptive function is identified
Measurement situation	Highly structured with clear boundaries; limited in terms of behavioral expression; often limited in terms of space usage; run in the laboratory facility	Allows for spontaneous activity; run in the natural or semi-natural settings
Effort required from the individual participant		When measurement done correctly, the individual often does not experience increased challenge related to the measurement
Scientific conduct of the researcher	Brings clear verification of the hypotheses; provides elegant publishable results; high risk of ecological validity fallacy	Provides rich behavioral data, placed within ecologically valid situations; vulnerability to ex-post interpretation
Effort required from the researcher	Main workload is allocated to preparation of the experiment and subsequent data analysis	Compared to a typical lab experiments, a lot of time and effort is invested in behavioral data coding and categorization
Time required to collect data	Relatively short	Relatively long

Brief examination of the characteristics of the ethological and experimental approaches suggests that they are neither in direct competition nor are they mutually interchangeable. Each has its strengths and limitations. As such, they should be treated as complementary.

Attempts to use the ethological approach have been made in a handful of recent research projects on the behavior of autistic children. One example is the study by Pegoraro et al. (2014), which appears not to have gained the recognition it deserves. The authors developed a detailed ethogram of the child’s behavior, specifically designed for autistic children. It allows experimenters to record 87 indices of behavior in the experimental condition. By analyzing the resulting ethogram, the authors were able to study the whole of the child’s behavior, including its complexity and its interpersonal and situational context. As such, the tool is useful for the holistic analysis of children’s behavior, though it should be mentioned that each experimental situation requires construction of a custom ethogram adapted to its specifics. One possible limitation of the ethogram might be its excessive level of detail, requiring a lot of processing work to describe and interpret each recorded behavior. Some of the simplified elements of the ethogram made their way to research on exploration in typically developing children and children with developmental disorders (Frostig et al., 2020; Kaur et al., 2015; Kawa & Pisula, 2010, 2013; Pisula, 2004).

While the ethological approach seems promising in terms of refining methods for measuring and analyzing behavior as a whole, some useful theoretical cues for designing such studies are offered by the theory of integrative levels. Incidentally, this theoretical framework has also been significantly underused. Its advantages along with potential experimental implementation rooted in the ethological approach to measurement of behavior are discussed below.

### Integrative Levels Theory and Research on Exploration

There is no doubt that the choice of the level of behavior recording and analysis determines later interpretation and conclusions about the aspect of functioning for which a given behavioral activity is considered to be a key indicator. Thus, if the indicator is locomotor activity, conclusions will be drawn about locomotor exploration. If the indicator is target fixation, the interpretation will tend towards visual exploration. As was already mentioned, this research perspective narrows the object of analysis, increasing the risk that any hypotheses explaining observed phenomena may lose their relevance for other behavioral contexts of analyzed behavior that eludes such analysis. The whole organism, rather than just eyes or hands, explores the surroundings, though at various phases of the exploration process a given form of behavior and a given organ may be the dominant one.

Attempts to find links between pervasive and individual (such as strictly defined eye or hand movements) characteristics of behavior encounter fundamental problems too often ignored by behavior scientists. We will illustrate this idea by outlining Feibleman’s (1954) theory of integrative levels and its implementation with respect to exploratory behavior (Pisula, 1998).

Feibleman (1954) presented a useful and clear summary of this important concept. We are selecting a few crucial points relevant for psychology.

1. "Each level organizes the level or levels below it plus one emergent quality." (p. 59). Each behavioral act may be described in terms of muscle reflexes, but for some behaviors these will be not enough. Purposive movements may include something more than just muscle reflexes.
2. "In any organization the higher level depends upon the lower." (p. 60). Destruction of the sensory-motor system disturbs behavior, but the reverse is not the case. Behavior disturbances do not cause damage to the sensory-motor system.
3. "For an organization at any given level, its mechanism lies at the level below and its purpose at the level above." (p. 61). This is obvious when we consider that an analysis moves from the whole to its parts. Nerve cells can tell us about mechanisms of brain functioning, but not about the purpose or function of the brain processes.
4. "It is impossible to reduce a higher level to the lower." (p. 62). To reduce a higher to a lower level means to lose the quality which emerged at this level. Therefore it is no longer the same phenomenon.

These descriptive points are supplemented by others which relate to explanation, especially of behavioral phenomena. Two of these are: (1) “The analysis of the phenomenon must be at the lowest level which will provide sufficient explanation.” (p. 63), and (2) “The reference of any organization must be to the highest level which its explanation requires.” (p. 64). That is to say: one cannot explain the phenomenon without bringing to the explanation elements belonging to the highest level of the phenomenon.

The implementation of the integrative levels theory to exploration in animals and humans was proposed by Pisula (1998). The point of departure here is the fact that Berlyne’s (1962) definition of exploratory behavior quoted towards the beginning of this paper does not capture the challenges faced by those who study that behavior. Thinking in terms of integrative levels leads us to the conclusion that both functions and mechanisms of behavior vary. Their variation depends on the level of integration. Below is a brief description of integrative levels of exploratory behavior put forward by Pisula (1998).

#### Taxes and Orienting Reflex

The term taxis implies a kind of invariant, reflex like automaticity of response that was, during the late 19th and early twentieth centuries, the basis of a search for rules and laws of behavioral reactions to external forces. Orienting reflex and taxis describe any turning of the body with reference to the position of a specific stimulus. In fact, orienting reflex is synonymous with taxis. It is triggered by a sudden, unexpected (for the organism) stimulus.

#### Perceptual Exploration

This refers to prolonged perception, by any sensory system, of a specific stimulation. This is a rather direct extension of the orienting response.

#### Locomotor Exploration

This refers to the situation in which the organism is moving within, or approaching a novel environment. It is also the most basic form of information-seeking, which can be described as controlled behavior (Pisula, 2001).

#### Investigatory Behavior

This includes various behavioral activities such as: interaction with the investigated object, manipulation of the object, investigation of a particular area (prolonged staring), etc. Investigatory responses possess the function of learning about the properties of the objects and their relationship to other elements in the environment. While perceptual exploration makes it possible to replenish the existing representation of an object or area with information about its stimulatory properties, manipulatory responses allow the individual to add information about the weight and structure of the object it explores. They also enable it to form contingencies between a given behavior and the environment. Both forms of information gathering depend on the ability to construct complex cognitive representations of objects. It is therefore the prerequisite of higher forms of information-seeking: knowledge-seeking at the cognitive level.

#### Cognitive Curiosity

We use this term in a more descriptive way than Berlyne (1962) did. It extends to include novelty-seeking and information and stimulation at the cognitive level. Berlyne was probably the first researcher to notice the close relationship between exploratory behaviors and cognitive activity and used the term “knowledge-seeking behavior”. He emphasized the symbolic character of knowledge acquired in this manner. Consequently, the informative value of that stimulation for that recipient will be higher than for a recipient not equipped with symbolic processing abilities. We should mention here once again the tenets of the theory of the integrative levels. It is obvious that symbolic behavior (knowledge-seeking) is dependent on the lower levels of behavior integration (e.g. exploration), and contains these lower-level components. However, this level of behavior cannot be reduced to lower levels. What it does, though, is take into account the integrative levels and the ecological approach (Gibson, 1988) that emphasizes the dependence of the stimulus’ meaning on the individual’s perceptual abilities.

When investigating a phenomenon as complex as behavior it is difficult to resist the temptation of reductionism in research procedures and conclusions. Today we know that the mind arises in the brain, which in turn consists of neurons, glial and other types of cells, and they in turn are made up of cell membranes built from proteins and lipids, etc. Most scientists will agree, however, that any attempt to explain mental phenomena purely through molecular interactions is bound to fail. In addition, neuroscientists have pointed out that a reductionist approach may lead to false conclusions (Krakauer et al., 2017). And although reductionism enjoyed some spectacular successes in the natural sciences and, to a lesser extent, psychology, there is nothing to suggest that it could be an effective approach to understanding and explaining human behavior. Feibleman (1954) was right to note that while cells are part of tissues, the latter are more than just a collection of similar cells; they have added value arising out of the relationships between those cells. An organism is more than a sum of its tissues and organs; a social group is not merely a sum of the characteristics of individuals that make it up, etc. The same is true of behavior: to understand it fully requires much more than even the most detailed knowledge about its components. Mating (animals) or dancing (humans) rituals consist of movements and sounds, but their meanings are revealed not through the physiological analysis of motor activity (though that knowledge is also necessary), but by seeing these behavioral acts in a wider situational and social context.

### Theoretical and Methodological Postulates for Psychological Studies Involving Actual Behavior

Psychology is the scientific study of mind and behavior, according to the American Psychological Association. In order to develop a successful paradigm for researching behavior and mind, it is a good idea to go back to the basics, i.e. the holistic analysis of behavior. As for exploratory behavior, we can demonstrate that behavioral analysis consistent with the premises of the theory of integrative levels should meet a number of conditions often ignored in today’s research. We believe that the following theoretical principles are crucial:

1. The behavior of an organism is not a sum of individual behavioral acts or responses of individual sensory effectors or analyzers. It is the property of the organism as a whole, realized by all resources available to the individual and at various integrative levels. The ultimate interpretation of behavior should be refer to the highest observed level of behavior regulation, in accordance with the theory of integrative levels (Feibleman, 1954).
2. An individual’s behavior occurs in a specific social context and actual physical environment. The requirement to ensure ecological validity of behavior measurement is fundamental for those who study behavior. Laboratory conditions, despite obvious advantages in terms of controlling the experimental setting, may generate information that inaccurately represents behavior in actual everyday circumstances. This may lead to situations where it is difficult to fully link laboratory data with behavior in the wild.
3. Complex organisms are characterized by high levels of individual differences. This is true of both interindividual and intraindividual differences. These differences may be manifested in the ways of achieving similar adaptive goals involving divergent uses of mechanisms present at various integrative levels.

The above principles may be implemented with the use of methods developed in mid-twentieth century in animal ethology, as well as modern tools assisting in the analysis of behavior, such as software for coding behavior records in video material (e.g. Friard & Gamba, 2016). While data derived from studies on more narrowly defined exploration processes (object exploration, visual exploration) are valuable, it seems unreasonable to give up on a much wider perspective, one that makes it possible to measure various components and aspects of behavior and attempt their holistic interpretation. In a more contemporary formulation, this proposal remains compatible with the notion of behavioral syndromes (Sih et al., 2004). A behavioral syndrome is a suite of correlated behaviors expressed either within a given behavioral context or across different contexts. As Sih proposed: behavioral syndromes could play a useful role as a central core in interdisciplinary studies that integrate genetics, neuroendocrine and developmental bases of behavior, and ecological consequences of behavior (p. 269). Other possible important behavioral carryovers emerged from attempts to explain apparently maladaptive behavior. In several cases, researchers (see Sih et al., 2004 for review) have suggested that an important general behavioral tendency has spilled over to produce inappropriate behavior in similar contexts or situations, making the concept of behavioral syndromes particularly relevant to analyses of certain clinical behavioral traits, such as autism.

Our suggestion is to look closely at the potential of a research approach combining achievements of animal and human ethology, integrative levels, and modern analytic methods available in the context of making audio-visual recordings of behavior and their qualitative and quantitative analysis. This approach draws inspiration from the framework that used to be called etho-experimental (Blanchard & Blanchard, 1988). We may summarize it by listing some basic principles of conducting research:

1. The setting in which the child’s behavior is recorded should allow for full expression of available behavioral repertoire, preferably with no restrictions on movement or contents of performed activities.
2. Recording techniques should ensure the capture of the full range of behavior demonstrated during the measurement.
3. Coding of behavior should follow the principles of creating an ethogram of the behavioral repertoire to later allow for complex, including sequential analysis of behavior.
4. Individual behavioral acts should be viewed from the perspective of the function they serve and in the context of chains of actions or complex forms of behavior of which they are part.
5. Behavior analysis should contain clear references to the behavior’s mechanisms and its adaptive function/goal.
6. Any interpretation of behavioral activity should refer to the level of behavior organization, taking into account the hierarchical structure of the levels of behavior integration/organization and avoiding excessive reductionism or over-interpretation.

An approach to behavior based on the analysis of its various aspects/manifestations can be particularly useful in research on how children on the autism spectrum learn about the environment, both physical and social. A variety of behaviors may have regulatory significance by reducing the level of arousal, which may be difficult to capture in experimental studies, where the measurement is based on much more narrowly defined indicators of behavior.

A study by L. Mottron et al. (2007), on lateral glances toward moving stimuli, may serve as a good example. Although the developed system of behavior coding did not go beyond looking at objects, it included their various manifestations in the child's contact with objects with different physical properties. The method used confirmed clinical observations indicating that these unusual behaviors, such as lateral glances in relation to moving objects, are more common among young autistic children than their typically developing peers. The authors suggest that these behaviors may reflect early attempts to address and/or regulate both excessive local information input and reduced movement perception. They also note that the study results can be treated as initial evidence for the need to consider the neural bases and development of atypical behaviors and their implications for intervention strategies. In our opinion, the extension of recording and analysis to spontaneous behavioral acts not directly related to the arranged experimental task has enriched this exciting study. A similar approach to the analysis of other unusual behaviors of children on the autism spectrum (including, for example, self-stimulating behavior) may provide valuable information in setting directions for further, more analytically, and theoretically advanced research.

Another field of study that may benefit from the approach proposed here is play behavior. A search in the ScienceDirect database based on the criteria ["spontaneous play" AND autism, years 2000–2022] returns 48 papers (search date 18 Dec 2022). Given the significance of play behavior for understanding the development of autistic children, this is a strikingly low score. Researchers sometimes attempt to propose an approach similar to the one presented here (Jefferies et al., 2018; Knickmeyer et al., 2008; Libby et al., 1998; Szabó, 2014; Vig, 2007), but this research line cannot break through to the mainstream of research in the area in question. It can be hypothesized that one possible reason for this effect is the methodological difficulty in addressing play behavior. The proposed solution is to implement a holistic approach based on complete behavior in ecologically valid experimental situations, as described in the earlier part of this paper.

It should be mentioned that all elements required to implement this approach to the study of exploration in children on the autism spectrum, are easily achievable. Modern technology for recording behavior is relatively affordable and easy to configure for the purposes of any given study. The theory of integrative levels and its potential implementations have been part of the system of knowledge since the middle of the twentieth century. There are also good examples of building ethograms of children’s behavior (e.g. Fragaszy et al., 2016; Pegoraro et al., 2014), as well as tools for precise coding and sophisticated analysis of behavior (e.g. Friard & Gamba, 2016). Granted, the approach proposed here requires a lot of effort from the researcher and is certainly time-consuming. However, we believe that such efforts would be rewarded by improving our understanding of the behavior of children, including autistic children. We find the idea of returning to the study of actual behavior in psychology (Baumeister et al., 2007; Doliński, 2018) really inspiring.

Although this paper focuses on the subject of exploratory behavior in children on the autism spectrum, we are convinced that the approach proposed here may have applications in other areas of research on human behavior.
